# Trajectories of precarious employment and the risk of myocardial infarction and stroke among middle-aged workers in Sweden: A register-based cohort study

**DOI:** 10.1016/j.lanepe.2022.100314

**Published:** 2022-02-03

**Authors:** Nuria Matilla-Santander, Carles Muntaner, Bertina Kreshpaj, Virginia Gunn, Johanna Jonsson, Lauri Kokkinen, Jenny Selander, Sherry L Baron, Cecilia Orellana, Per-Olof Östergren, Tomas Hemmingsson, David H. Wegman, Theo Bodin

**Affiliations:** a Unit of Occupational Medicine, Institute of Environmental Medicine, Karolinska Institutet, Stockholm, Sweden; b Lawrence S. Bloomberg Faculty of Nursing and Division of Social and Behavioral Sciences, Dalla Lana School of Public Health, University of Toronto, Toronto, ON, Canada; c Lawrence S. Bloomberg Faculty of Nursing, University of Toronto & MAP Centre for Urban Health Solutions, Li Ka Shing Knowledge Institute, Toronto, ON, Canada; d Faculty of Social Sciences, Tampere University, Tampere, Finland; e Barry Commoner Center for Health and the Environment, Queens College, City University of New York, New York City, USA; f Social Medicine and Global Health, Department of Clinical Sciences Malmö, Lund University, Sweden; g Department of Public Health Sciences, Stockholm University, Sweden; h University of Massachusetts Lowell, Lowell, USA; i Centre for Occupational and Environmental Medicine, Stockholm Region, Stockholm, Sweden

**Keywords:** Group-based model trajectories, Cardiovascular, Collective bargaining agreements, Income, Temporary agency work, Multiple job holding, Unstable employment

## Abstract

**Background:**

The aim is to identify trajectories of precarious employment (PE) over time in Sweden to examine associations of these with the subsequent risk of myocardial infarction (MI) and stroke.

**Methods:**

This is a nation-wide register-based cohort study of 1,583,957 individuals aged 40 to 61 years old residing in Sweden between 2003-2007. Trajectories of PE as a multidimensional construct and single PE components (contractual employment relationship, temporariness, income levels, multiple job holding, probability of coverage by collective agreements) were identified for 2003-2007 by means of group-based model trajectories. Risk Ratios (RR) for MI and stroke according to PE trajectories were calculated by means of generalized linear models with binomial family.

**Findings:**

Adjusted estimates showed that constant PE and borderline PE trajectories increased the risk of MI (RR: 1·08, CI95%:1·05-1·11 and RR:1·13, CI95%: 1·07-1·20 respectively) and stroke (RR:1·14, CI95%: 1·10-1·18 and HR:1·24, CI95%: 1·16-1·33 respectively) among men. A higher risk of stroke in men was found for the following unidimensional trajectories: former agency employees (RR:1·32, CI95%:1·04-1·68); moving from high to a low probability of having collective agreements (RR: 1·10, CI95%:1·01-1·20). Having constant low or very low income was associated to an increased risk of MI and Stroke for both men and women.

**Interpretation:**

The study findings provide evidence that PE increases the risk of stroke and possibly MI. It highlights the importance of being covered by collective bargaining agreements, being directly employed and having sufficient income levels over time.

**Funding:**

The Swedish Research Council for Health, Working Life and Welfare, no. 2019-01226.

**Research in context**

**Evidence before this study**

We searched PubMed without language restrictions for observational studies published until October 2021 reporting associations between precarious employment (PE) or any of its components and myocardial infarction (MI, fatal/non-fatal) and stroke (fatal/non-fatal) for any country. We used the MeSH terms “(precarious employment OR precarious work OR precarious job OR employment patterns OR non-standard employment OR income OR agency employment OR temporary employment OR union* OR collective bargaining agreements OR multiple job-holding OR temporary agency employment) AND (stroke OR myocardial infarction OR cardiovascular)”. Evidence suggest that determined single components of PE (mostly temporary employment and low income) are associated with MI or stroke. Although, in our search, we could not find any study exploring the risk of MI or stroke that was using a multidimensional measurement of PE or based in trajectories and neither exploring specific single components of PE such as multiple job holding or coverage by collective bargaining agreements. Moreover, previous evidence exploring the cardiovascular risk associated with PE has several limitations, including, evidence based in cross-sectional studies that are susceptible to recognized biases (length-biased sampling, recall bias, common method bias, etc...) and reverse causation, use of one-time point exposure measurements, and use of only unidimensional measurements of PE.

**Added value of this study**

This is the first study to examine the risk of myocardial infarction and stroke associated with trajectories of precarious employment (PE) as a multidimensional construct and its components. We do so by means of a register-based and nation-wide cohort study of 1,583,957 individuals in Sweden (2003-2017). Using group-based trajectory modelling, the study identifies trajectories of workers using their scores of overall PE and single components. The study findings show that to be in a trajectory of constant PE increases the risk of stroke by 24% in men. Further, three component trajectories related to PE increase the risk of stroke in men: former agency employment (by 32%), moving from high to low probability of coverage by collective agreements (by 10%) and being in constant very low-income levels (by 19%).

**Implications of all the available evidence**

Our study is based on a comprehensive approach to PE which includes an analysis of multiple components of PE and their combined effect. By using such approach, it is possible to provide specific direction to initiatives addressing the effects of PE on health. Our findings highlight the importance of being covered by collective bargaining agreements, being directly employed and having sufficient income levels over time.

Alt-text: Unlabelled box

## Introduction

Although mortality from cardiovascular disease (CVD) in Sweden has been on a continuous decline since the 1970s, CVDs remain the major cause of morbidity and mortality in Sweden and globally.^1^ While this decline is linked to improvements in prevention strategies and advances in treatment of CVDs, these benefits have not been equally distributed across socio-demographic groups.^2^ Previous studies have shown that low socio-economic position may increase the risk of CVD in a similar manner as biomedical risk factors (e.g., hypertension, dyslipidaemia).^3^ Therefore, since overall population health cannot improve if segments of the population do not benefit from advances in prevention, understanding the influence of social inequities in the development of CVDs is key.^4^ In this regard, low income and education level have been consistently associated with an increased risk of CVD, while the association with certain employment and working conditions (i.e., physical strenuous work, non-standard employment arrangements) have been inconclusive, possibly as a result of inconsistent definitional, theoretical, and methodological approaches, as briefly outlined next.^5^, ^6^, ^7^, ^8^

Increasing evidence suggests that precarious employment (PE, employment that includes several low-quality characteristics, such as low income, lack of workplace rights, temporariness, low unionization) is associated with an array of unfavourable health outcomes.^9^, ^10^, ^11^ However, there is still a knowledge gap regarding the effects of PE on CVDs.

Previous evidence exploring the CVD risk associated with PE conditions has some limitations. First, most studies are based on one-dimensional measures of PE (that is considering only one of the dimensions/characteristics that makes employment precarious). For instance, part-time work (characterized by income insufficiency)^12^ and temporary employment (characterized by employment insecurity),^13^ have been associated with several cardiovascular risk factors and cardiovascular diseases. The limitation of using only one-dimensional measures of PE is the possible miss-classification of individuals.^7^^,^^14^ Therefore, a more comprehensive approach to PE, including an analysis of multiple components of PE and their combined effect, would reduce the likelihood of miss-classification. Moreover, within this approach, the study of single components of PE provides important information for guiding initiatives addressing the effects of PE on health.

Second, most previous studies exploring associations between non-standard employment and cardiovascular disease are cross-sectional. Therefore, these studies are susceptible to recognized biases of such studies (length-biased sampling, recall bias, common method bias, etc...) and cannot rule out reverse causation (the possibility that due to a CVD diagnosis individuals have less opportunities of maintaining a non-precarious situation in the labour market).^15^ Third, most existing prospective studies use one-time point exposure measurements. These measurements do not allow the study of mobility patterns of workers. This is especially relevant, since previous studies have found that workers exposed to income volatility have an increased risk of CVD mortality^16^ and those shifting from temporary to permanent employment have a lower risk of death (including cardiovascular mortality).^17^

Against this background, this study aimed to identify trajectories of PE and its components over time in Sweden in order to examine associations of these with the subsequent risk of myocardial infarction (MI) and stroke in workers in Sweden.

## Methods

### Study design and data collection

This is a register study based on the Swedish Work, Illness, and Labour-market Participation (SWIP) cohort. The SWIP cohort consists of a total population cohort of individuals registered in Sweden in 2005 (approx. 5·4 million individuals), aged 16 to 65 years old. For the purposes of the present study, 1,583,957 individuals are included in the study population. This is a cohort, where individuals aged 40 to 61 years old residing in Sweden started to be followed between 2003-2005 until the end of 2017. This cohort uses multiple data sources that were linked by Statistics Sweden:

(i) Longitudinal Integration Database for Health Insurance and Labor Market Studies register (LISA): contains socio-demographic and employment data.
(ii) National patient register: includes dates of admission and diagnosis using the International Classification of Diagnoses (ICD-codes) from inpatient and specialized outpatient care.
(iii) The Swedish prescribed drug register: contains prescribed and purchased medications classified according to the Anatomical, Therapeutic, Chemical classification system (ATC) codes of pharmaceuticals, including dates of purchase.
(iv) Cause of Death register: contains dates and cause (ICD-codes) of death.
(v) Swedish multi-generation register: contains connections between index persons (i.e., individuals in our cohort) and their biological or adoptive parents.

Exclusion criteria for the study were: (i) incomplete information for measuring the exposure variable during at least three years between 2003-2007 (we only included individuals who had at least 3 years information for measuring their PE score and not the whole 5 years to avoid the exclusion of individuals with worse employment trajectories, such as frequent switching between employment and unemployment, and therefore reduce the likelihood of survivor or immortality bias), (ii) solo and non-solo self-employed during 2003-2007 (to avoid their miss-classification as precariously employed), (iii) yearly employer-based income of <100 Swedish Krona (SEK) during 2003-2007, (iv) death, emigration or immigration during 2003-2007, (v) age <40 or >62 in 2003, (vi) to have received old-age or early pension benefits >10000 SEK during 2003-2007 (to avoid miss-classifying individuals leaving the labour market into PE), and (vii) MI (ICD-10 codes: I21-I22) or stroke (ICD-10 codes: I61-I63-I64) diagnosis between 1997-2007 (to minimize the risk of reverse causation). See Figure S1 for the flow chart of the total population included.

### Study variables

#### Exposure variable: trajectories of PE as a multidimensional construct and its components (2003-2007)

The construction of this variable followed two steps:

First. Scoring of PE and its components: we used version 2.0 of the Swedish Register-based Operationalization of Precarious Employment (SWE-ROPE).^18^ SWE-ROPE consists of five components (contractual employment insecurity, temporariness, multiple job-holding, income level, coverage under collective bargaining agreements) covering the three dimensions (employment insecurity, income inadequacy and lack of rights and protection) of PE as identified by Kreshpaj et al.^9^ Individuals were assigned a yearly score for each component:

- Contractual employment insecurity score: ranges from -1 to 0, as follows: -1 (agency employed) and 0 (directly employed).
- Temporariness score: ranges from -2 to 0, as follows: -2 (unstable employment) and 0 (stable employment). Measured as a change in employers in the past 2 years.
- Multiple job holding score: ranges from -2 to 0, as follows: -2 (multiple jobs and multiple sectors), -1 (multiple jobs), 0 (no multiple jobs). Measured as having one or more employers in the current year and the economic sector of these employers.
- Income level score: ranges from -2 to 2, as follows: -2 (income lower than 60% of the median, being very low income levels), -1 (income between 60 and 80% of the median, being low income levels), 0 (income between 81 and 120% of the median, being medium income levels), 1 (income between 121 and 199% of the median, being high income levels), 2 (income higher than 200% of the median, being high income levels). Measured based on work-related income, work-related social insurance benefits (parental benefits, sickness benefits and related sources), and unemployment benefits.
- Probability of unionisation score: based on the probability of coverage under collective bargaining agreements and ranges from -2 to 0, as follows: -2 (less than 70%), -1 (from 71 to 90%), 0 (more than 90%). This is based on the probability of certain groups (company size, ownership sector, economic sector, sex) being covered by occupational pension.

The sum of the score of each of these five items resulted in a summative score ranging from -9 to 2 (PE score). A detailed description and discussion regarding the operationalization can be found in a previous paper.^5^ We considered an individual to be in PE when the total score was < -3. When an individual has a PE score in between -2 and -1 they are in borderline PE (are at the limit of being considered precariously employed).

Second. Group Based Model Trajectories (GBMT): we constructed trajectories using the score of the five components of PE and the total PE score:

- Model search: we used censored normal regression models for all the components and total PE score, except when constructing trajectories for the components “contractual employment insecurity” and “temporariness”, for which we applied a logistic model (category 0 = score 0, category 1 = score –1 or -2). Given availability of five-time points measurements, we started by testing a quadratic model for all groups. The final number of groups was determined based on the Bayesian and Akaike Information Criterion (BIC and AIC), the proportion of cohort members in each class, and changes in the patterns of the trajectories. After identifying the optimal number of groups, the level of the polynomial for each group was adjusted until a parameter estimate in the highest function had a p-value <0.01.
- Model diagnostics: we assessed the models following the four criteria described by Nagin et al:^19^ (i) average probability of assignment for each group is 0.7 or higher, (ii) odds of correct classification are 5.0 or higher, (iii) the proportion of a sample assigned to a certain group is close to the proportion estimated from the model, and (iv) 99% confidence intervals of the estimated proportion are reasonably narrow. See Table S1 for these diagnostics.

We also constructed the trajectories for only men and only women, and for two age groups to further investigate if patterns would differ across these groups.

#### Outcome variables: cardiovascular diseases (2008-2017)

First incidence of non-fatal MI (ICD-10 codes: I21-I22) or stroke (ICD-10 codes: I61-I63-I64) or death due to MI or stroke during 2008-2017.

#### Other variables

We obtained the minimal sufficient set of variables for adjustment by drawing the causal assumptions using a Directed Acyclic Graph (DAG) (Figure S2). Confounders included were:

• Socio-demographic characteristics: age, country of birth (Sweden, other than Sweden), educational level (primary/secondary education, higher education), family composition (single, single with children, cohabiting, cohabiting with children), sex (female, male), a proxy of one parent's socio-economic status during an individual's childhood (manual, non-manual worker, farmer/self-employed, unknown) measured in 1960, 1970, 1980, 1990. Missing data in this variable was categorized as “unknown” and in order to do not exclude individuals not born in Sweden (80% of workers not born in Sweden had missing data for this variable). Confounders were measured for the year when the first exposure measurement was available for everyone (2003, 2004 or 2005).
• Cardiovascular risk factors: pharmaceuticals purchased during exposure measurement (available from 2005 to 2007) for treating diabetes mellitus (ATC codes: A10A, A10B), dyslipidaemia (ATC codes: C10A, C10B) or hypertension (ATC codes: C03A, C03B, C03C, C03D, C03D, C03E, C03X, C07A, C07B, C07C, C07D, C07E, C07F, C08C, C08D, C08E, C08G, C09A, C09B, C09C, C09D) as a proxy for cardio-metabolic health conditions.

Moreover, when exploring associations between trajectories of single PE components and MI and stroke, we adjusted the models for the remaining trajectories of single PE components.

Further, we calculated the share of workers in each precarious trajectory according to occupational titles (at the second digit-level) using the Standard Swedish Occupational Classification (ssyk-96).^20^

### Statistical analysis

First, we calculated accumulated incidences of MI and stroke during 2008-2017. Next, we calculated crude and adjusted risk ratios (RR) with 95% confidence intervals (CIs) for MI and stroke according to PE trajectories by means of generalized linear models with binomial family and log link. All analyses were stratified by sex given previous evidence showing that (i) the health effects of being in PE differ across women and men^6^ and (ii) both the incidence of MI and stroke and prevalence of PE differ between men and women (see Tables 1 and 2).

**Table 1:** Baseline characteristics of the individuals included in this study according to precarious employment trajectories (n= 1,583,957).

	Female (%)	Age 42-50 (%)	Higher education (%)	Not Swedish born (%)	Private sector (%)
**Overall**	50·85	35·13	20·72	10·41	52·74
**Precarious employment trajectories**					
*Constant non-PE*	44·07	33·36	26·07	9·45	52·49
*From non-PE to PE*	46·94	38·88	13·56	10·64	65·24
*Constant borderline precarious*	67·94	37·32	10·14	12·06	46·54
*Constant precarious*	58·93	43·41	12·01	14·83	73·97
**Components of Precarious Employment**					
**Contractual relation insecurity trajectories**					
*Constant directly employed*	50·83	35·10	20·72	10·39	52·63
*Former agency employed*	55·76	46·48	19·59	17·49	88·98
**Temporariness trajectories**					
*Constant not temporary*	54·17	32·18	20·72	10·13	45·59
*From temporary to not temporary*	44·28	41·71	22·17	11·69	68·58
*From not temporary to temporary*	42·18	41·94	19·06	10·34	69·40
**Multiple job holding trajectories**					
*Constant not MJH*	50·93	35·09	20·54	10·39	52·67
*Constant MJH*	46·13	37·51	31·58	11·98	56·58
**Income level trajectories**					
*Constant high income*	27·45	34·59	40·70	7·71	62·59
*Constant good income*	50·40	34·99	16·61	10·75	53·57
*Constant low income*	79·86	37·26	7·08	13·16	37·06
*Constant very low income*	85·40	31·27	6·70	11·41	44·32
**Probability of coverage by collective agreements trajectories**					
*Constant high CBA*	51·62	34·70	21·15	10·35	49·70
*From low to high CBA*	31·40	44·06	16·24	9·20	83·23
*From high to low CBA*	52·22	42·11	14·17	10·37	98·08
*Constant low CBA*	32·17	38·29	14·41	13·58	98·13

Abbreviations: PE (precarious employment), MJH (multiple job holding), CBA (collective bargaining agreement). Note: higher education refers to tertiary education of 3 or more years.

**Table 2:** Cases and accumulated incidence per 100 persons of myocardial infarction and stroke (2008-2017) according to precarious employment trajectories (2003-2007), n= 1,583,957.

	MYOCARDIAL INFARCTION				STROKE			
	Men		Women		Men		Women	
	Cases	cumulative incidence	cases	cumulative incidence	cases	cumulative incidence	cases	cumulative incidence
**Overall**	29953	3·85	9685	1·20	20054	2·58	11547	1·43
**Precarious employment trajectories**								
*Constant non-PE*	21432	3·75	4996	1·11	14191	2·48	6159	1·37
*From non-PE to PE*	1973	3·85	542	1·19	1354	2·64	662	1·46
*Constant borderline precarious*	5297	4·19	3623	1·35	3612	2·86	4135	1·54
*Constant precarious*	1251	4·35	524	1·27	897	3·12	591	1·43
**Components of Precarious Employment**								
**Contractual relation insecurity trajectories**								
*Constant directly employed*	29885	3·85	9651	1·20	19988	2·57	11511	1·43
*Former agency employed*	68	3·28	34	1·30	66	3·19	36	1·38
**Temporariness trajectories**								
*Constant not temporary*	19989	3·97	7540	1·27	13460	2·67	8873	1·49
*From temporary to not temporary*	5311	3·67	1167	1·01	3589	2·48	1478	1·28
*From not temporary to temporary*	4653	3·58	978	1·03	3005	2·31	1196	1·26
**Multiple job holding trajectories**								
*Constant not MJH*	29408	3·85	9557	1·20	19671	2·57	11380	1·43
*Constant MJH*	545	3·88	128	1·06	383	2·72	167	1·39
**Income level trajectories**								
*Constant high income*	9342	3·23	873	0·80	6360	2·20	1341	1·22
*Constant good income*	17649	4·13	5013	1·16	11499	2·69	5944	1·37
*Constant low income*	2540	4·75	3033	1·43	1890	3·54	3361	1·59
*Constant very low income*	422	4·92	766	1·53	305	3·56	901	1·80
**Probability of coverage by collective agreements trajectories**								
*Constant high CBA*	27459	3·84	9199	1·21	18343	2·57	10949	1·44
*From low to high CBA*	610	3·80	59	0·80	406	2·53	92	1·25
*From high to low CBA*	759	3·72	264	1·18	539	2·64	306	1·37
*Constant low CBA*	1125	4·12	163	1·26	766	2·80	200	1·54

Abbreviations: PE (precarious employment), MJH (multiple job holding), CBA (collective bargaining agreement).

**Table 3:** Adjusted hazard ratios of myocardial infarction and stroke according to precarious employment trajectories by sex, n= 1,583,957.

	MYOCARDIAL INFARCTION				STROKE			
	MEN		WOMEN		MEN		WOMEN	
	aHR	CI95%	aHR	CI95%	aHR	CI95%	aHR	CI95%
**Precarious employment trajectories**								
*Constant non-PE*	ref	ref	ref	ref	ref	ref	ref	ref
*From non-PE to PE*	0·99	0·95-1·04	0·98	0·90-1·08	1·03	0·98-1·09	1·03	0·95-1·11
*Constant borderline PE*	1·08	1·05-1·11	1·06	1·02-1·11	1·14	1·10-1·18	1·07	1·02-1·11
*Constant PE*	1·13	1·07-1·20	1·06	0·97-1·16	1·24	1·16-1·33	1·04	0·95-1·13
**Contractual relation insecurity trajectories**								
*Constant directly employed*	ref	ref	ref	ref	ref	ref	ref	ref
*Former agency employed*	0·90	0·72-1·14	1·18	0·85-1·65	1·32	1·04-1·68	1·02	0·74-1·42
**Temporariness trajectories**								
*Constant not temporary*	ref	ref	ref	ref	ref	ref	ref	ref
*From unstable to stable employment*	1·00	0·97-1·03	0·93	0·87-0·99	1·03	0·98-1·06	0·98	0·93-1·04
*From stable to unstable employment*	0·98	0·95-1·02	0·93	0·86-0·99	0·96	0·92-1.00	0·96	0·90-1·02
**Multiple job holding trajectories**								
*Constant not MJH*	ref	ref	ref	ref	ref	ref	ref	ref
*Constant MJH*	1·03	0·95-1·12	1·05	0·88-1·25	1·05	0·95-1·16	1·07	0·92-1·25
**Income level trajectories**								
*Constant good income*	ref	ref	ref	ref	ref	ref	ref	ref
*Constant high income*	0·88	0·86-0·9	0·85	0·79-0·92	0·90	0·87-0·93	0·99	0·94-1·06
*Constant low income*	1·08	1·04-1·12	1·11	1·06-1·16	1·19	1·15-1·27	1·10	1·05-1·15
*Constant very low income*	1·11	1·01-1·22	1·04	0·97-1·13	1·17	1·05-1·33	1·09	1·02-1·17
**Probability of coverage by collective agreements trajectories**								
*Constant high CBA*	ref	ref	ref	ref	ref	ref	ref	ref
*From low to high CBA*	1·04	0·96-1·13	0·77	0·60-0·99	1·05	0·94-1·15	0·99	0·81-1·22
*From high to low CBA*	1·01	0·94-1·09	0·99	0·87-1·12	1·10	1·01-1·20	0·98	0·88-1·10
*Constant low CBA*	1·00	0·94-1·06	0·97	0·83-1·13	1·00	0·92-1·07	0·99	0·87-1·15

Note: aHR are adjusted for continuous age, educational level, family composition, country of birth, purchased medication for treating diabetes, hypertension and dyslipidaemia during exposure measurement, parental socio-economic status of the parents during childhood of study participants and contractual relation insecurity, temporariness, multiple job holding, income level and probability of coverage by collective bargaining agreements trajectories.

Abbreviations: PE (precarious employment), MJH (multiple job holding), CBA (collective bargaining agreement).

Further, we conducted several sensitivity analyses:

(i) We calculated the adjusted estimates excluding fatal MI and stroke cases to test if the risk differed according to fatal and non-fatal events.
(ii) We stratified the adjusted estimates according to:
  a. age at baseline (42-50 and 51-65 years old) to further investigate if some groups had higher risks.
  b. income at baseline (dichotomized into low- and high-income levels) for further exploring the distinct effect of income from other components of PE.

Data management and statistical analysis was conducted with STATA 16.

### Role of the funding source

The funding source of the study had no role in study design, data collection, data analysis, data interpretation, or writing of the report.

## Results

### Trajectories of PE and its components

**Figure 1:**
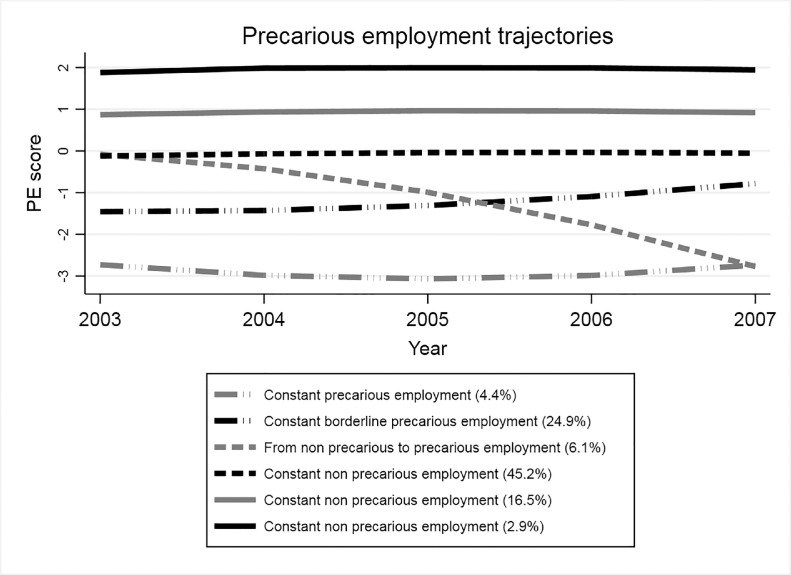
Trajectories of precarious and non-precarious employment (2003-2007) in middle aged workers in Sweden (n= 1,583,957). Note: PE (PE score). An individual is in precarious employment when the total score is < -3. The three trajectories corresponding to non-precarious employment are merged for the analysis into one trajectory “constant non precarious employment”.

This study identified six different trajectories for the combined PE scoring, three of which involved PE at any point: (i) moving from non-precarious to PE (6·1%), (ii) constant borderline PE (24·9%), and (iii) constant PE (4.4%) (Figure 1).

**Figure 2:**
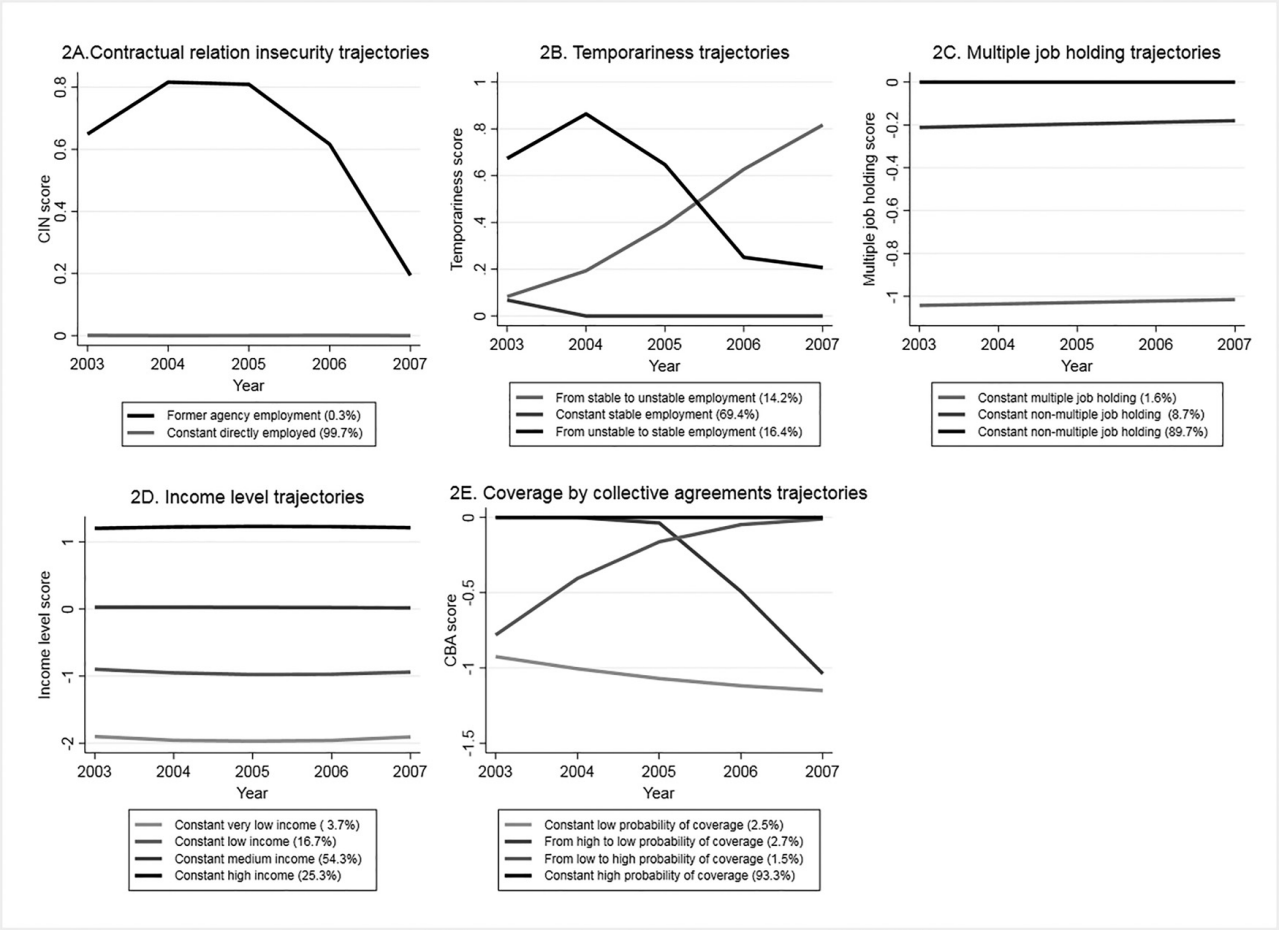
Trajectories of components of precarious employment (2003-2007) in middle aged workers in Sweden (n= 1,583,957). Note: 2A. CIN (Contractual relation insecurity) score (0 = directly employed, 1= temporary agency employed), 2B Temporariness score (0 = stable employment, 1= unstable employment), 2C. Multiple job holding score (0 = non multiple job holder, -1= multiple job holder, -2= multiple job holder in multiple sectors), the two trajectories corresponding to non-multiple job holding are merged for the analysis into “constant non-multiple job holding”, 2D. Income levels score (-2= income <60% of the median, -1= income 60-80% of the median, 0= income 81-120% of the median, 1= income 121-199% of the median, 2= income >200% of the median), 2E. CBA (probability of coverage by collective agreements) score (-2 = less than 70%, -1= from 71 to 90%, 0= more than 90%).

Figure 2 shows the trajectories of each of the five components of PE. In terms of contractual relationship, we describe a trajectory of former agency employed workers (0·30%) (Figure 2A). Next, we found two trajectories that involved unstable employment (moving from or to unstable employment) (Figure 2B). In regards multiple job holding, 1·6% remained constant multiple job holders (Figure 2C). Constant low (16·7%) and very low income (3·7%) trajectories were also found (Figure 2D). Finally, we identified a trajectory of being in constant low probability of being covered by collective bargaining agreements (2·5%) and, another one of individuals moving from high to low probability of coverage (2·7%) (Figure 2E).

Trajectories conducted among women and men separately, and age-groups showed similar patterns (data not shown).

### Socioeconomic composition of the trajectories of PE

Constant PE and borderline PE trajectories were more frequent among women, lower educated workers, not Swedish born and among workers from the private sector (Table 1).

Also, constant PE and borderline PE were frequent among agricultural, fishery skilled workers and labourers, labourers in mining, construction, manufacturing and transport, sales and services elementary occupations. Shifting from non-precarious to PE was more common among office clerks (in the case of men) and customer services clerks (in the case of women) (Table S2).

### Incidence of MI and stroke

Incidence of MI and stroke was higher among men in constant PE and borderline PE, and among those moving from non-PE to PE (Table 2). In terms of trajectories within the components of PE, MI and stroke incidence was higher among constant low or very low income, constant low probability of coverage by collective agreements and constant not temporary trajectories.

### The associations between PE trajectories and MI and stroke

Adjusted estimates showed that constant PE and borderline PE increased the risk of MI (RR: 1·08, CI95%:1·05-1·11 and RR:1·13, CI95%: 1·07-1·20) and stroke (RR:1·14, CI95%: 1·10-1·18 and HR:1·24, CI95%: 1·16-1·33 respectively) among men (Table 3).

In terms of trajectories of components of PE, those that increase the risk of stroke among men were former agency employed (RR:1·32, CI95%:1·04-1·68), to move from high to low probability of coverage by collective agreements (RR: 1·10, CI95%:1·01-1·20) and to be in constant low and very low income (HR: 1·17, CI95%: 1·05-1·33) (Table 3).

The trajectories of components of PE that increased the risk of MI among men were only those in constant low or very low income.

Constant multiple job holding did not show associations with any of the outcomes, and trajectories involving unstable employment showed a slightly protective effect for stroke among men (HR: 0·96, CI95%:0·92-1·00) and for MI among women (HR: 0·93, CI95%: 0·86-0·99).

Unadjusted estimates can be found in Table S3. Adjusted estimates stratified according to age groups (42-50 and 51-65 years old at baseline) were similar (Table S4 and S5). Next, estimates for fatal and non-fatal cases of MI and stroke, also showed similar patterns, although, being in constant low or very low-income trajectories increased the risk of fatal MI and stroke (Table S6 and S7).

Adjusted estimates for the trajectories of components of PE stratified according to income levels at baseline showed no increased risk in the case of MI (Table S8). The risk of stroke was increased among men in low-income levels that were former agency employed (HR: 1·46, CI95%:1·10-1·92), and that moved from high to low probability of coverage by collective agreements (HR: 1·14, CI95%: 1·03-1·26) (Table S9). The risk of MI and stroke was increased in constant PE and borderline PE across low income earners and among medium income earners only for stroke (table S10 and S11).

## Discussion

### Main findings

This study found that to be in constant PE or borderline PE increases the risk of stroke and possibly MI among men. The trajectories of components of PE that increased the risk of stroke were being former agency employed, shifting from high to low probability of coverage by collective agreements, and to be in constant low income. In the case of MI, the only PE component that increased the risk was constant low income.

### Interpretation

To be continuously in PE and borderline PE increases the risk of stroke in men (by 24 and 14%, respectively). These results are in line with previous studies linking non-standard employment arrangements, job insecurity and income volatility with poorer cardiovascular health.^16^^,^^21^ This study adds to the literature the multidimensional, objective and working-life approach for measuring PE.

Trajectories of components of PE that increased the risk of stroke among men were shifting from high to low probability of coverage by collective agreements (by 10%), being former agency employed (by 32%) and being in continuous low-income levels (by 19-21%). Therefore, the increased risk of stroke observed among precarious trajectories may be explained by these three components of PE.

The increased risk observed among those shifting from high to low probability of coverage by collective agreements supports the relevant role of labour unions on bargaining for decent health and safety standards for workers, and also employment and working conditions, such as higher wage and benefit standards, working hour limits, or workplace hazards protections.^22^ Moreover, although Nordic labour models are characterized by high levels of unionization, the share of Swedish workers who are members of a trade union has dropped in the last decade from 80% to 70%, especially among blue-collar workers.^23^ Therefore, our results, provide moderate evidence that low probability of coverage by collective agreement (a proxy measurement of unionization) may be associated with serious health implications. So, interventions that aim to reduce the health effects of PE should also consider coverage by collective agreements.

Moreover, the findings associated with the trajectory involving temporary agency employment, show that even though individuals were former agency employed workers, their risk of stroke was increased by 32% compared to those that were constantly in direct employment.

Low and very low-income levels trajectories were consistently associated with an increased risk of stroke. Income is a well-established risk factor for cardiovascular diseases. Interestingly, previous studies have found an increased risk of MI associated with income volatility,^16^^,^^24^ but our study did not find fluctuating income trajectories. This could be a result of the Swedish welfare regime, the yearly resolution of the data, the restriction to middle-aged workers in our study and perhaps, the share of workers with fluctuating trajectories was really low and was not well captured using GBTM.

PE trajectories may increase the risk of stroke through several pathways; here we note increased exposure to excessive physical work demands, which would change intravascular turbulence and arterial wall shear stress, inducing inflammatory processes, leading to atherosclerosis.^25^ Also, PE workers may be exposed to irregular working hours to a higher extent, which could alter circadian rhythms, affecting glucose and lipid metabolism, inflammation, and autonomic nervous system regulation, and increasing the risk of atherosclerosis, dyslipidaemia, and insulin resistance.^26^ Next, workers in PE may be more exposed to stressful work environments (because of effort/reward imbalances^27^ and increased job strain^28^). The work environment stress could increase the risk of stroke through a neuro-immunologic pathway (increased activity of the amygdala, a neural tissue involved in the response to stress, which, in turn could increase arterial inflammation).^29^ Also, PE is often associated with unhealthy lifestyles and increased weight, hypertension, and diabetes.^30^ All of these conditions could act as mediators in the causal pathway between PE and cardiovascular disorders. Future studies conducting mediation analysis will allow a better understanding of the mechanisms through which PE affects cardiovascular health.

To be in constant PE or borderline PE also increased the risk of MI among men (by 13 and 9%, respectively). Although, when exploring the risk associated to the components of PE, only trajectories of constant low or very income increased the risk for MI. A larger proportion of MI take place under the age of 50 (5%) compared to stroke (3·5%). When applying our strict exclusion criteria regarding previous disease this could lead to more severe selection bias / healthy survivor bias for MI, leading to a comparatively underestimation of the results.

The SWE-ROPE^18^ scores multiple job holding and instability as very negative for the employment relationship quality. In our study, the trajectories of these items indicated no association (in the case of constant multiple job holding) or even a protective effect (in the case of the trajectories involving unstable employment) with the studied disorders. The protective effects of the trajectories involving unstable employment could be explained because it is not possible to differentiate between voluntary change of employers. Unstable employment is measured as changing employers over three years, but this could also include individuals changing employers as an improvement in their careers. Given the age groups included in our study (40 to 61), this possibility is quite plausible.

In our study, we found constant PE or borderline PE is more common among occupations related to agriculture and fishery, mining, construction, manufacturing, transport and sales and services. These occupations are associated with specific workplace risks, which also have been associated with cardiovascular diseases. Therefore, future studies should explore if and how these workplace risks interact with PE and affect cardiovascular health.

Finally, this study did not find an increased risk of MI or stroke among women exposed to precarious trajectories. This could be explained by several reasons. Probably PE is increasing the risk of MI and stroke through lifestyle factors, and it is well known that men have poorer lifestyle factors compared to women. Also, there is the possibility of an interaction between precarious employment and specific occupational risk factors among male-dominated occupations (i.e., agriculture and fishery, mining, construction.”

### Limitations and strengths

This study has several limitations. First, we cannot rule out a healthy worker effect that may have introduced a healthy survivor bias and therefore underestimated the estimates. Our study population includes individuals that need to be “healthy” enough to work under PE conditions. We tried to minimize this bias by including individuals who have information of their PE score for at least three years (allowing that for one or two years they may not have information on the exposure due to for example unemployment). Moreover, we could not account for lifestyle behaviours in our study. Although we have adjusted for the level of education which is often highly correlated with lifestyle risk factors. We have also adjusted the estimates by pharmaceuticals for treating diabetes, hypertension, and dyslipidaemia (during the exposure measurement), which are often highly correlated with unhealthy lifestyles such as diet and physical activity. So, adjustment by these variables may have reduced the odds of residual confounding by lifestyle behaviours. Also, the measurement of PE did not include some components, such as length of the contract, involuntary part-time, or ability to exercise rights, which may have better captured the impact of PE on cardiovascular health. Next, some individuals may have changed their precarious status during the follow-up (2008-2017). This would probably imply a shift from PE to non-PE and therefore an underestimation of the effect observed.

About GBTM methods, GBTM allow to study the potential heterogeneity in the development of PE or non-PE status all over the years by identifying different “subpopulations” characterized by distinct trajectories. On the other, GBTM are population average approaches and are not optimal for finding trajectories that are less common.

Regarding strengths, this study is based on register data, which allows for an objective measurement of PE (not based in self-reported data). Moreover, since population registers have high validity, low attrition rates and are nation-wide,^31^ the results of this study are representative of middle-aged workers (salaried) in Sweden and the likelihood of selection bias is reduced. Next, the measurement of outcomes based on national patient registers is of high quality, as these registers are of high quality and coverage (outpatient and inpatient care).

Also, the prospective design of the study makes it possible to study PE trajectories as risk factors for MI and stroke, and the exclusion of individuals with previous cardiovascular events reduces the likelihood of reverse causation.

## Conclusions

This study found that to be in constant PE or borderline PE increases the risk of stroke and probably MI among men. Moreover, it highlights the importance of being covered by collective bargaining agreements, being directly employed and having sufficient income levels over time.

## Declaration of interests

None declared.

## Appendix Supplementary materials

Image, application 1
