# Supplementary material for: Trajectories of precarious employment and the risk of myocardial infarction and stroke among middle-aged workers in Sweden: A register-based cohort study
Source: Lancet Reg Health Eur. 2022 Feb 3;15:100314. doi: 10.1016/j.lanepe.2022.100314 (PMC8829810; doi:10.1016/j.lanepe.2022.100314)
Supplement: Supplementary file 1 [file mmc1.docx]

**Supplementary Material**

**Trajectories of precarious employment and the risk of myocardial infarction and stroke among middle-aged workers in Sweden: a register-based cohort study.**

**Figure S1. Flow chart of the total population included. (Page 2)**

**Table S1. Diagnostics of the group-based trajectories final models. (Page 3)**

**Figure S2. Directed Acyclic Graph of the association between precarious employment trajectories and risk of cardiovascular disease. (Page 4)**

**Table S2. Trajectories of precarious employment according to occupation. (Pages 5-6)**

**Table S3. Crude risk ratios of myocardial infarction and stroke according to employment trajectories. (Page 7)**

**Table S4. Adjusted risk ratios for myocardial infarction according to precarious employment trajectories and age-groups at baseline. (Page 8)**

**Table S5. Adjusted risk ratios for stroke according to precarious employment trajectories and age-groups at baseline. (Page 9)**

**Table S6. Adjusted risk ratios of myocardial infarction (non-fatal and fatal cases) according to employment trajectories. (Page 10)**

**Table S7. Adjusted risk ratios of stroke (non-fatal and fatal cases) according to employment trajectories. (Page 11)**

**TableS8. Adjusted risk ratios of myocardial infarction according to employment trajectories and stratified according to income levels at baseline. (Page 12)**

**Table S9. Adjusted risk ratios of stroke according to employment trajectories and stratified according to income levels at baseline. (Page 13)**

**Table S10. Adjusted risk ratios for stroke according to precarious employment trajectories and income levels at baseline. (Page 14)**

**Table S11. Adjusted risk ratios for stroke according to precarious employment trajectories and income levels at baseline. (Page 15)**

**Figure S1. Flow chart of the total population included.**


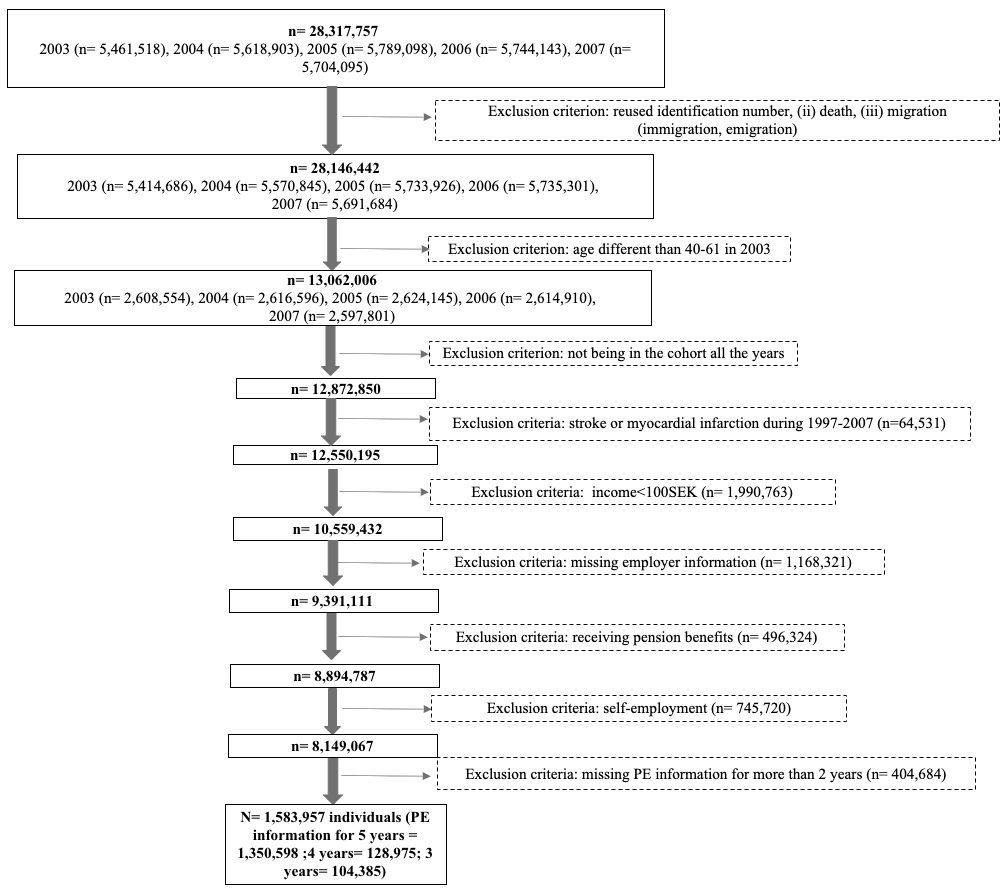


Abbreviations: PE (Precarious Employment)

**Table S1. Diagnostics of the group-based trajectories final models.**

| **Models** | **Group** | **Estimated proportion from the trajectory model** | **99% confidence interval for the estimated proportion** | **Proportion by posterior probability-based classification** | **Average posterior probability** | **Odds of correct classification** |
| --- | --- | --- | --- | --- | --- | --- |
| **Precarious employment**  **trajectories** | **1** | 4·5 | 4·5-4·6 | 4·4 | 93·6 | 310·375 |
|  | **2** | 23·6 | 23·5-23·7 | 24·9 | 87·9 | 23·51715927 |
|  | **3** | 6·4 | 6·4-6·5 | 6·1 | 87·2 | 99·6328125 |
|  | **4** | 46·7 | 46·6-46·8 | 45·2 | 94·5 | 19·61008371 |
|  | **5** | 15·7 | 15·6-15·8 | 16·5 | 89·3 | 44·81213167 |
|  | **6** | 2·8 | 2·8-2·9 | 2·9 | 97·6 | 1411·714286 |
| **Contractual relation insecurity trajectories** | **1** | 0·3 | 0·30-0·32 | 0·30 | 99·2 | 41209·33333 |
|  | **2** | 99·6 | 99·6-99·7 | 99·7 | 99·9 | 4·012048193 |
| **Temporariness trajectories** | **1** | 15·1 | 15·03-15·2 | 14·2 | 91·7 | 62·11864677 |
|  | **2** | 67·9 | 67·8-68 | 69·4 | 97·9 | 22·03934357 |
|  | **3** | 16·9 | 16·8-17 | 16·4 | 92·6 | 61·53094515 |
| **Multiple job holding trajectories** | **1** | 1·9 | 1·8-1·9 | 1·65 | 84·1 | 273·0953327 |
|  | **2** | 13·1 | 13·1-13·2 | 8·69 | 94·1 | 105·8001035 |
|  | **3** | 84·8 | 84·8-84·9 | 89·7 | 94·7 | 3·202741189 |
| **Income level trajectories** | **1** | 3·6 | 3·6-3·7 | 3·7 | 98·4 | 1646·833333 |
|  | **2** | 16·7 | 16·6-16·8 | 16·7 | 97·7 | 211·8825827 |
|  | **3** | 53·8 | 53·7-53·9 | 54·3 | 98·3 | 49·65514979 |
|  | **4** | 25·7 | 25·6-25·8 | 25·3 | 99·6 | 719·8715953 |
| **Collective bargaining agreement trajectories** | **1** | 2·6 | 2·5-2·6 | 2·54 | 97·1 | 1254·31565 |
|  | **2** | 2·8 | 2·8-2·9 | 2·70 | 96·02 | 837·5039483 |
|  | **3** | 15·7 | 15·5-15·9 | 1·48 | 94·3 | 88·83104257 |
|  | **4** | 92·9 | 92·8-92·9 | 93·28 | 99·6 | 19·03013994 |

**Figure S2. Directed Acyclic Graph of the association between precarious employment trajectories and risk of cardiovascular disease.**


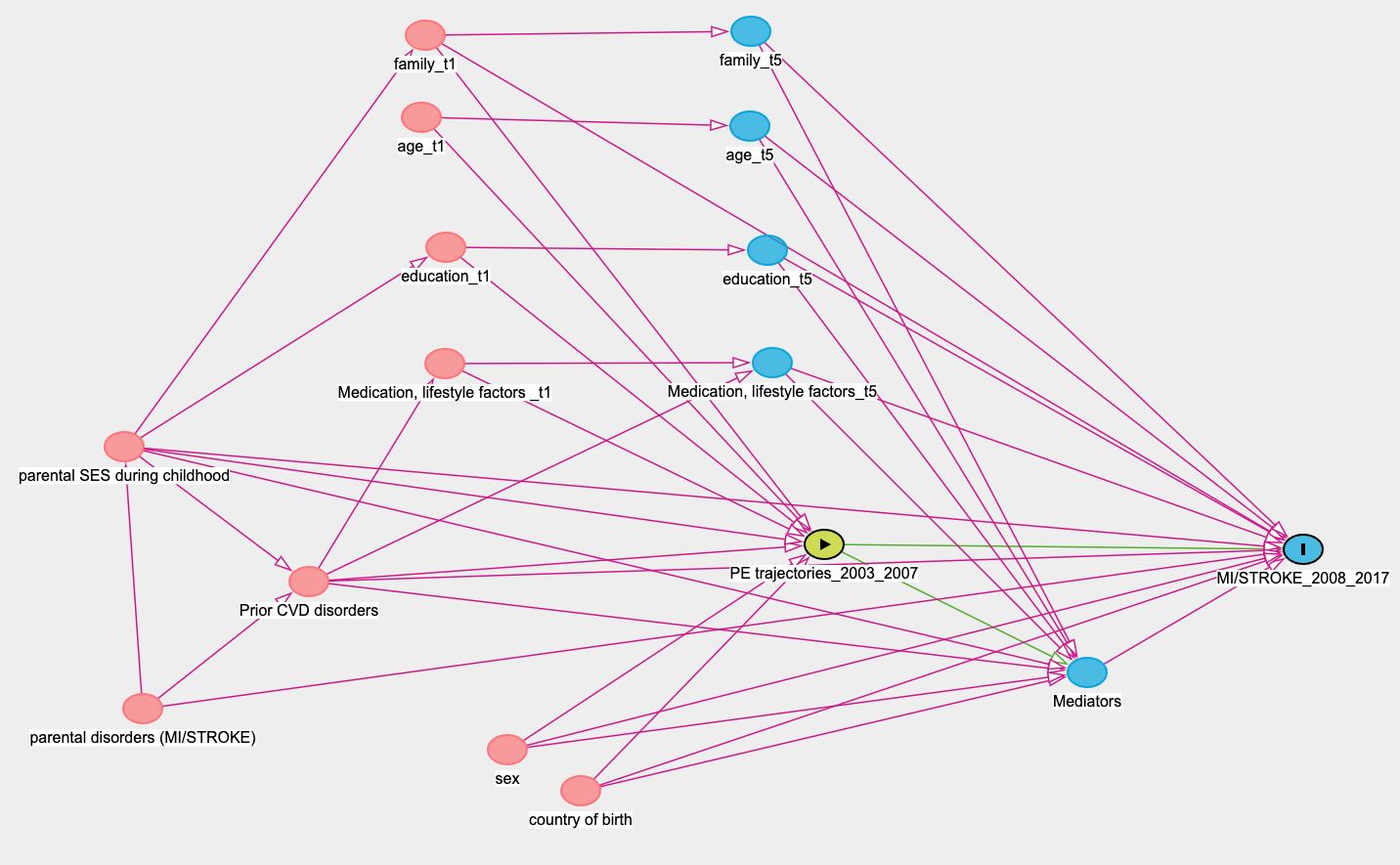


Minimal sufficient adjustment sets for estimating the total effect of precarious trajectories (PE_trajectories) on MI/stroke:

- Medication, lifestyle factors _t1, Prior CVD disorders, age_t1, country of birth, education_t1, family_t1, parental SES during childhood, sex
- Medication, lifestyle factors _t1, Prior CVD disorders, age_t1, country of birth, education_t5, family_t1, parental SES during childhood, sex
- Medication, lifestyle factors _t1, Prior CVD disorders, age_t5, country of birth, education_t1, family_t1, parental SES during childhood, sex
- Medication, lifestyle factors _t1, Prior CVD disorders, age_t5, country of birth, education_t5, family_t1, parental SES during childhood, sex
- Medication, lifestyle factors_t5, Prior CVD disorders, age_t1, country of birth, education_t1, family_t1, parental SES during childhood, sex
- Medication, lifestyle factors_t5, Prior CVD disorders, age_t1, country of birth, education_t5, family_t1, parental SES during childhood, sex
- Medication, lifestyle factors_t5, Prior CVD disorders, age_t5, country of birth, education_t1, family_t1, parental SES during childhood, sex
- Medication, lifestyle factors_t5, Prior CVD disorders, age_t5, country of birth, education_t5, family_t1, parental SES during childhood, sex

Abbreviations: MI (Myocardial infarction), CVD (Cardiovascular disease), t1: individual characteristic at entry in the cohort, _t5: individual characteristic at baseline. Mediators could include working hazards, medication, stressful work, mental disorders, behavioral risk factors.

**Table S2. Trajectories of precarious employment according to occupation (2-digit level. ssyk-96 codes)**

|  | **MEN** | | | | **WOMEN** | | | |
| --- | --- | --- | --- | --- | --- | --- | --- | --- |
| **Occupation (2-digit level. ssyk-96 codes)** | **Constant non-PE** | **From non-PE to PE** | **Constant borderline PE** | **Constant PE** | **Constant non-PE** | **From non-PE to PE** | **Constant borderline PE** | **Constant PE** |
| **Total** | 73·47 | 6·59 | 16·25 | 3·69 | 55·96 | 5·64 | 33·28 | 5·12 |
| **Armed forces** | 88·10 | 7·41 | 2·32 | 2·18 | 88·73 | 1·41 | 5·63 | 4·23 |
| **Legislators and senior officials** | 84·68 | 4·08 | 7·80 | 3·45 | 84·98 | 3·29 | 9·08 | 2·66 |
| **Corporate managers** | 91·82 | 3·40 | 3·88 | 0·90 | 89·62 | 3·84 | 5·55 | 1·00 |
| **Managers of small enterprises** | 70·16 | 5·21 | 17·70 | 6·92 | 69·97 | 6·56 | 18·38 | 5·09 |
| **Physical. mathematical and engineering science professionals** | 90·35 | 2·63 | 6·31 | 0·71 | 85·06 | 3·88 | 9·92 | 1·13 |
| **Life science and health professionals** | 93·51 | 1·76 | 4·13 | 0·60 | 82·58 | 3·26 | 12·76 | 1·41 |
| **Teaching professionals** | 83·08 | 4·34 | 9·84 | 2·75 | 81·01 | 3·93 | 12·71 | 2·35 |
| **Other professionals** | 83·71 | 4·58 | 9·16 | 2·55 | 77·36 | 5·27 | 14·90 | 2·47 |
| **Physical and engineering science associate professionals** | 82·97 | 4·23 | 11·63 | 1·17 | 70·16 | 6·09 | 21·71 | 2·04 |
| **Life science and health associate professionals** | 77·37 | 8·04 | 12·57 | 2·02 | 71·91 | 4·36 | 21·29 | 2·43 |
| **Teaching associate professionals** | 69·44 | 5·98 | 18·75 | 5·83 | 66·43 | 3·69 | 27·21 | 2·67 |
| **Other associate professionals** | 79·25 | 5·24 | 12·56 | 2·95 | 60·33 | 7·04 | 28·80 | 3·83 |
| **Office clerks** | 56·72 | 20·42 | 18·88 | 3·97 | 51·76 | 8·40 | 34·02 | 5·81 |
| **Customer services clerks** | 49·81 | 11·25 | 30·09 | 8·85 | 37·35 | 12·51 | 39·25 | 10·90 |
| **Personal and protective services workers** | 61·30 | 6·79 | 24·13 | 7·79 | 41·44 | 4·01 | 49·59 | 4·96 |
| **Models. salespersons and demonstrators** | 54·80 | 9·03 | 28·67 | 7·50 | 26·60 | 9·99 | 47·83 | 15·58 |
| **Skilled agricultural and fishery workers** | 46·87 | 8·29 | 31·55 | 13·29 | 27·51 | 9·21 | 39·19 | 24·09 |
| **Extraction and building trades workers** | 66·02 | 7·24 | 22·29 | 4·45 | 40·55 | 6·74 | 43·32 | 9·39 |
| **Metal. machinery and related trades workers** | 71·65 | 6·94 | 19·15 | 2·26 | 58·77 | 6·41 | 31·38 | 3·45 |
| **Precision. handicraft. craft printing and related trades workers** | 68·48 | 8·17 | 21·02 | 2·33 | 46·51 | 9·79 | 38·10 | 5·61 |
| **Other craft and related trades workers** | 54·64 | 8·22 | 28·56 | 8·58 | 37·50 | 8·00 | 44·56 | 9·94 |
| **Stationary-plant and related operators** | 86·44 | 4·62 | 8·06 | 0·88 | 75·62 | 8·09 | 15·06 | 1·24 |
| **Machine operators and assemblers** | 70·54 | 7·98 | 19·32 | 2·17 | 53·24 | 8·63 | 33·81 | 4·32 |
| **Drivers and mobile-plant operators** | 58·00 | 9·97 | 24·68 | 7·36 | 47·27 | 10·03 | 32·02 | 10·67 |
| **Sales and services elementary occupations** | 39·88 | 11·35 | 37·84 | 10·94 | 13·06 | 6·10 | 67·65 | 13·18 |
| **Agricultural. fishery and related labourers** | 34·11 | 8·53 | 39·53 | 17·83 | 20·60 | 6·03 | 47·74 | 25·63 |
| **Labourers in mining. construction. manufacturing and transport** | 52·41 | 5·36 | 38·63 | 3·59 | 29·47 | 5·68 | 58·24 | 6·60 |
| **Missing values** | 56·30 | 5·67 | 24·72 | 13·32 | 31·71 | 6·67 | 34·57 | 27·06 |

**Note:** PE (Precarious Employment), Ssyk-96 codes (Swedish standard classification of occupations).

**Table S3. Crude risk ratios of myocardial infarction and stroke according to precarious employment trajectories.**

|  | **MYOCARDIAL INFARCTION** | | | | | | | **STROKE** | | | | | |
| --- | --- | --- | --- | --- | --- | --- | --- | --- | --- | --- | --- | --- | --- |
|  | **MEN** | | | | **WOMEN** | | | **MEN** | | | **WOMEN** | | |
|  | **RR** | | **CI95%** | | **RR** | **CI95%** | | **RR** | **CI95%** | | **RR** | **CI95%** | |
| **Precarious employment trajectories** |  | |  |  |  |  |  |  |  |  |  |  |  |
| ***Constant non-PE*** | ref | | | | ref | | | ref | | | ref | | |
| ***From non-PE to PE*** | 1·05 | | 1·01 | 1·10 | 1·12 | 1·03 | 1·23 | 1·10 | 1·04 | 1·16 | 1·12 | 1·03 | 1·21 |
| ***Constant borderline PE*** | 1·17 | | 1·13 | 1·20 | 1·24 | 1·19 | 1·30 | 1·22 | 1·18 | 1·26 | 1·16 | 1·11 | 1·20 |
| ***Constant precarious*** | 1·22 | | 1·15 | 1·29 | 1·23 | 1·13 | 1·35 | 1·34 | 1·25 | 1·43 | 1·14 | 1·05 | 1·24 |
| **Contractual relation insecurity trajectories** |  | |  |  |  |  |  |  |  |  |  |  |  |
| ***Constant direct employed*** | ref | | | | ref | | | ref | | | ref | | |
| ***Former agency employed*** | 0·92 | 0·73 | | 1·16 | 1·23 | 0·88 | 1·72 | 1·37 | 1·08 | 1·74 | 1·07 | 0·77 | 1·48 |
| **Temporariness trajectories** |  |  | |  |  |  |  |  |  |  |  |  |  |
| ***Constant not temporary*** | ref | | | | ref | | | ref | | | ref | | |
| ***From temporary to not temporary*** | 1·01 | | 0·98 | 1·04 | 0·93 | 0·88 | 0·99 | 1·03 | 0·99 | 1·07 | 1·00 | 0·94 | 1·05 |
| ***From not temporary to temporary*** | 0·99 | | 0·96 | 1·02 | 0·95 | 0·89 | 1·02 | 0·96 | 0·93 | 1·01 | 0·99 | 0·93 | 1·05 |
| **Multiple job holding trajectories** |  | |  |  |  |  |  |  |  |  |  |  |  |
| ***Constant not MJH*** | ref | | | | ref | | | ref | | | ref | | |
| ***Constant MJH*** | 0·99 | | 0·91 | 1·08 | 1·04 | 0·87 | 1·24 | 1·02 | 0·92 | 1·13 | 1·07 | 0·92 | 1·25 |
| **Income level trajectories** |  | |  |  |  |  |  |  |  |  |  |  |  |
| ***Constant good income*** | ref | | | | ref | | | ref | | | ref | | |
| ***Constant high income*** | 0·78 | | 0·76 | 0·80 | 0·69 | 0·64 | 0·74 | 0·81 | 0·79 | 0·84 | 0·90 | 0·84 | 0·95 |
| ***Constant low income*** | 1·12 | | 1·07 | 1·16 | 1·24 | 1·19 | 1·30 | 1·26 | 1·20 | 1·32 | 1·16 | 1·12 | 1·21 |
| ***Constant very low income*** | 1·13 | | 1·03 | 1·24 | 1·18 | 1·09 | 1·27 | 1·22 | 1·09 | 1·36 | 1·15 | 1·08 | 1·24 |
| **Collective bargaining agreement trajectories** |  | |  |  |  |  |  |  |  |  |  |  |  |
| ***Constant high CBA*** | ref | | | | ref | | | ref | | | ref | | |
| ***From low to high CBA*** | 1·03 | | 0·95 | 1·12 | 0·78 | 0·60 | 1·01 | 1·04 | 0·94 | 1·14 | 1·00 | 0·81 | 1·23 |
| ***From high to low CBA*** | 1·01 | | 0·94 | 1·09 | 1·02 | 0·90 | 1·16 | 1·10 | 1·01 | 1·20 | 0·99 | 0·88 | 1·11 |
| ***Constant low CBA*** | 1·00 | | 0·94 | 1·06 | 0·99 | 0·85 | 1·16 | 0·99 | 0·92 | 1·06 | 1·02 | 0·89 | 1·17 |

Note: RR adjusted for age at baseline (continuous). Abbreviations: PE (Precarious Employment), MJH (multiple job holders), CBA (collective bargaining agreement).

**Table S4. Adjusted risk ratios for myocardial infarction according to precarious employment trajectories and baseline age-groups**

|  | **MEN** | | | | | | | **WOMEN** | | | | | |
| --- | --- | --- | --- | --- | --- | --- | --- | --- | --- | --- | --- | --- | --- |
|  | **42-50** | | | | **51-65** | | | **42-50** | | | **51-65** | | |
|  | **RR** | | **CI95%** | | **RR** | **CI95%** | | **RR** | **CI95%** | | **RR** | **CI95%** | |
| **Precarious employment trajectories** |  | |  |  |  |  |  |  |  |  |  |  |  |
| ***Constant non-PE*** | ref | | | | ref | | | ref | | | ref | | |
| ***From non-PE to PE*** | 0·95 | | 0·86 | 1·05 | 1·00 | 0·95 | 1·05 | 1·09 | 0·90 | 1·33 | 0·96 | 0·87 | 1·07 |
| ***Constant borderline PE*** | 1·09 | | 1·02 | 1·16 | 1·08 | 1·04 | 1·12 | 1·06 | 0·95 | 1·18 | 1·07 | 1·02 | 1·12 |
| ***Constant precarious*** | 1·17 | | 1·04 | 1·30 | 1·10 | 1·03 | 1·18 | 1·08 | 0·89 | 1·32 | 1·05 | 0·95 | 1·17 |
| **Contractual relation insecurity trajectories** |  | |  |  |  |  |  |  |  |  |  |  |  |
| ***Constant directly employed*** | ref | | | | ref | | | ref | | | ref | | |
| ***Former agency employed*** | 1·09 | 0·74 | | 1·59 | 0·81 | 0·60 | 1·10 | 1·08 | 0·51 | 2·26 | 1·22 | 0·84 | 1·77 |
| **Temporariness trajectories** |  |  | |  |  |  |  |  |  |  |  |  |  |
| ***Constant not temporary*** | ref | | | | ref | | |  | | |  | | |
| ***From temporary to not temporary*** | 0·99 | | 0·94 | 1·07 | 1·00 | 0·97 | 1·04 | 0·92 | 0·80 | 1·06 | 0·93 | 0·86 | 0·99 |
| ***From not temporary to temporary*** | 0·95 | | 0·88 | 1·02 | 0·99 | 0·95 | 1·03 | 0·94 | 0·81 | 1·09 | 0·91 | 0·85 | 0·99 |
| **Multiple job holding trajectories** |  | |  |  |  |  |  |  |  |  |  |  |  |
| ***Constant not MJH*** | ref | | | | ref | | | ref | | | ref | | |
| ***Constant MJH*** | 1·15 | | 0·97 | 1·37 | 1·00 | 0·91 | 1·10 | 1·15 | 0·79 | 1·66 | 1·03 | 0·84 | 1·26 |
| **Income level trajectories** |  | |  |  |  |  |  |  |  |  |  |  |  |
| ***Constant good income*** | ref | | | | ref | | | ref | | | ref | | |
| ***Constant high income*** | 0·89 | | 0·84 | 0·95 | 0·88 | 0·85 | 0·91 | 0·71 | 0·58 | 0·87 | 0·88 | 0·81 | 0·96 |
| ***Constant low income*** | 1·14 | | 1·04 | 1·25 | 1·06 | 1·02 | 1·11 | 1·12 | 1·00 | 1·25 | 1·11 | 1·06 | 1·17 |
| ***Constant very low income*** | 1·27 | | 1·04 | 1·55 | 1·06 | 0·95 | 1·18 | 1·20 | 0·99 | 1·46 | 1·03 | 0·95 | 1·12 |
| **Collective bargaining agreement trajectories** |  | |  |  |  |  |  |  |  |  |  |  |  |
| ***Constant high CBA*** | ref | | | | ref | | | ref | | | ref | | |
| ***From low to high CBA*** | 1·03 | | 0·88 | 1·21 | 1·04 | 0·95 | 1·15 | 0·84 | 0·51 | 1·37 | 0·75 | 0·56 | 1·02 |
| ***From high to low CBA*** | 1·08 | | 0·93 | 1·23 | 0·99 | 0·91 | 1·08 | 0·87 | 0·65 | 1·17 | 1·03 | 0·90 | 1·18 |
| ***Constant low CBA*** | 0·97 | | 0·85 | 1·11 | 1·01 | 0·94 | 1·08 | 1·03 | 0·72 | 1·46 | 0·95 | 0·80 | 1·13 |

Note: RR are adjusted for continuous age, educational level, family composition, country of birth, purchased medication for treating diabetes, hypertension and dyslipidaemia during exposure measurement, parental socio-economic status of the parents during childhood of study participants and contractual relation insecurity, temporariness, multiple job holding, income level and probability of coverage by collective bargaining agreements trajectories.

Abbreviations: PE (Precarious Employment), MJH (multiple job holders), CBA (collective bargaining agreement).

**Table S5. Adjusted risk ratios for stroke according to precarious employment trajectories and baseline age-groups**

|  | **MEN** | | | | | | | **WOMEN** | | | | | |
| --- | --- | --- | --- | --- | --- | --- | --- | --- | --- | --- | --- | --- | --- |
|  | **42-50** | | | | **51-65** | | | **42-50** | | | **51-65** | | |
|  | **RR** | | **CI95%** | | **RR** | **CI95%** | | **RR** | **CI95%** | | **RR** | **CI95%** | |
| **Precarious employmen trajectories** |  | |  |  |  |  |  |  |  |  |  |  |  |
| ***Constant non-PE*** | ref | | | | ref | | | ref | | | ref | | |
| ***From non-PE to PE*** | 1·13 | | 0·99 | 1·27 | 1·00 | 0·94 | 1·07 | 1·16 | 0·97 | 1·39 | 1·00 | 0·92 | 1·10 |
| ***Constant borderline PE*** | 1·10 | | 1·01 | 1·20 | 1·15 | 1·09 | 1·18 | 1·06 | 0·95 | 1·17 | 1·07 | 1·03 | 1·12 |
| ***Constant precarious*** | 1·21 | | 1·03 | 1·39 | 1·24 | 1·15 | 1·33 | 1·01 | 0·82 | 1·20 | 1·05 | 0·96 | 1·16 |
| **Contractual relation insecurity trajectories** |  | |  |  |  |  |  |  |  |  |  |  |  |
| ***Constant directly employed*** |  | | | |  | | |  | | |  | | |
| ***Former agency employed*** | 1·43 | 0·93 | | 2·18 | 1·28 | 0·95 | 1·71 | 1·11 | 0·57 | 2·14 | 0·99 | 0·68 | 1·44 |
| **Temporariness trajectories** |  |  | |  |  |  |  |  |  |  |  |  |  |
| ***Constant not temporary*** |  | | | |  | | |  | | |  | | |
| ***From temporary to not temporary*** | 1·06 | | 0·98 | 1·16 | 1·02 | 0·97 | 1·06 | 1·02 | 0·90 | 1·15 | 0·97 | 0·91 | 1·04 |
| ***From not temporary to temporary*** | 1·07 | | 0·98 | 1·17 | 0·93 | 0·89 | 0·98 | 1·06 | 0·92 | 1·21 | 0·94 | 0·88 | 1·01 |
| **Multiple job holding trajectories** |  | |  |  |  |  |  |  |  |  |  |  |  |
| ***Constant not MJH*** |  | | | |  | | |  | | |  | | |
| ***Constant MJH*** | 1·00 | | 0·78 | 1·27 | 1·06 | 0·95 | 1·18 | 0·83 | 0·56 | 1·21 | 1·13 | 0·95 | 1·33 |
| **Income level trajectories** |  | |  |  |  |  |  |  |  |  |  |  |  |
| ***Constant good income*** |  | | | |  | | |  | | |  | | |
| ***Constant high income*** | 0·94 | | 0·87 | 1·02 | 0·89 | 0·87 | 0·93 | 0·81 | 0·69 | 0·96 | 1·03 | 0·96 | 1·10 |
| ***Constant low income*** | 1·20 | | 1·07 | 1·35 | 1·19 | 1·12 | 1·25 | 1·08 | 0·97 | 1·19 | 1·11 | 1·06 | 1·17 |
| ***Constant very low income*** | 1·42 | | 1·11 | 1·83 | 1·12 | 0·99 | 1·26 | 0·94 | 0·77 | 1·15 | 1·13 | 1·04 | 1·22 |
| **Collective bargaining agreement trajectories** |  | |  |  |  |  |  |  |  |  |  |  |  |
| ***Constant high CBA*** |  | | | |  | | |  | | |  | | |
| ***From low to high CBA*** | 1·04 | | 0·85 | 1·28 | 1·04 | 0·93 | 1·17 | 0·95 | 0·62 | 1·45 | 1·01 | 0·80 | 1·28 |
| ***From high to low CBA*** | 1·16 | | 0·98 | 1·38 | 1·08 | 0·97 | 1·19 | 1·02 | 0·80 | 1·32 | 0·97 | 0·86 | 1·11 |
| ***Constant low CBA*** | 0·92 | | 0·77 | 1·10 | 1·01 | 0·94 | 1·10 | 0·87 | 0·61 | 1·25 | 1·03 | 0·88 | 1·20 |

Note: RR are adjusted for continuous age, educational level, family composition, country of birth, purchased medication for treating diabetes, hypertension and dyslipidaemia during exposure measurement, parental socio-economic status of the parents during childhood of study participants and contractual relation insecurity, temporariness, multiple job holding, income level and probability of coverage by collective bargaining agreements trajectories.

Abbreviations: PE (Precarious Employment), MJH (multiple job holders), CBA (collective bargaining agreement).

**Table S6. Adjusted risk ratios for fatal and non-fatal myocardial infarction according to precarious employment trajectories.**

|  | **FATAL MYOCARDIAL INFARCTION** | | | | | | | **NON-FATAL MYOCARDIAL INFARCTION** | | | | | |
| --- | --- | --- | --- | --- | --- | --- | --- | --- | --- | --- | --- | --- | --- |
|  | **MEN** | | | | **WOMEN** | | | **MEN** | | | **WOMEN** | | |
|  | **RR** | | **CI95%** | | **RR** | **CI95%** | | **RR** | **CI95%** | | **RR** | **CI95%** | |
| **Precarious employmen trajectories** |  | |  |  |  |  |  |  |  |  |  |  |  |
| ***Constant non-PE*** | ref | | | | ref | | | ref | | | ref | | |
| ***From non-PE to PE*** | 0·89 | | 0·77 | 1·02 | 0·96 | 0·73 | 1·26 | 1·01 | 0·96 | 1·06 | 0·99 | 0·90 | 1·09 |
| ***Constant borderline PE*** | 1·30 | | 1·19 | 1·41 | 1·09 | 0·95 | 1·25 | 1·06 | 1·02 | 1·09 | 1·06 | 1·01 | 1·11 |
| ***Constant precarious*** | 1·16 | | 0·99 | 1·36 | 0·87 | 0·64 | 1·17 | 1·13 | 1·06 | 1·20 | 1·07 | 0·98 | 1·18 |
| **Contractual relation insecurity trajectories** |  | |  |  |  |  |  |  |  |  |  |  |  |
| ***Constant directly employed*** | ref | | | | ref | | | ref | | | ref | | |
| ***Former agency employed*** | 0·87 | 0·43 | | 1·74 | 0·72 | 0·18 | 2·89 | 0·91 | 0·71 | 1·17 | 1·23 | 0·87 | 1·75 |
| **Temporariness trajectories** |  |  | |  |  |  |  |  |  |  |  |  |  |
| ***Constant not temporary*** | ref | | | | ref | | | ref | | | ref | | |
| ***From temporary to not temporary*** | 0·94 | | 0·86 | 1·03 | 0·67 | 0·54 | 0·84 | 1·01 | 0·98 | 1·05 | 0·95 | 0·89 | 1·02 |
| ***From not temporary to temporary*** | 0·86 | | 0·78 | 0·95 | 0·72 | 0·57 | 0·90 | 1·00 | 0·96 | 1·03 | 0·94 | 0·88 | 1·01 |
| **Multiple job holding trajectories** |  | |  |  |  |  |  |  |  |  |  |  |  |
| ***Constant not MJH*** | ref | | | | ref | | | ref | | | ref | | |
| ***Constant MJH*** | 1·16 | | 0·92 | 1·47 | 1·02 | 0·56 | 1·87 | 1·01 | 0·92 | 1·11 | 1·05 | 0·87 | 1·27 |
| **Income level trajectories** |  | |  |  |  |  |  |  |  |  |  |  |  |
| ***Constant good income*** | ref | | | | ref | | | ref | | | ref | | |
| ***Constant high income*** | 0·81 | | 0·75 | 0·88 | 0·75 | 0·57 | 0·98 | 0·88 | 0·86 | 0·91 | 0·87 | 0·80 | 0·94 |
| ***Constant low income*** | 1·45 | | 1·31 | 1·61 | 1·10 | 0·95 | 1·27 | 1·03 | 0·99 | 1·08 | 1·11 | 1·06 | 1·17 |
| ***Constant very low income*** | 1·46 | | 1·16 | 1·85 | 1·31 | 1·06 | 1·61 | 1·07 | 0·96 | 1·19 | 1·01 | 0·93 | 1·10 |
| **Collective bargaining agreement trajectories** |  | |  |  |  |  |  |  |  |  |  |  |  |
| ***Constant high CBA*** | ref | | | | ref | | | ref | | | ref | | |
| ***From low to high CBA*** | 1·09 | | 0·86 | 1·38 | 0·44 | 0·14 | 1·39 | 1·03 | 0·95 | 1·13 | 0·80 | 0·62 | 1·05 |
| ***From high to low CBA*** | 1·09 | | 0·88 | 1·34 | 1·06 | 0·72 | 1·55 | 1·00 | 0·93 | 1·08 | 0·98 | 0·86 | 1·12 |
| ***Constant low CBA*** | 0·91 | | 0·77 | 1·08 | 1·16 | 0·76 | 1·78 | 1·01 | 0·95 | 1·08 | 0·94 | 0·80 | 1·11 |

Note: RR are adjusted for continuous age, educational level, family composition, country of birth, purchased medication for treating diabetes, hypertension and dyslipidaemia during exposure measurement, parental socio-economic status of the parents during childhood of study participants and contractual relation insecurity, temporariness, multiple job holding, income level and probability of coverage by collective bargaining agreements trajectories.

Abbreviations: PE (Precarious Employment), MJH (multiple job holders), CBA (collective bargaining agreement).

**Table S7. Adjusted risk ratios for fatal and non-fatal stroke according to precarious employment trajectories.**

|  | **FATAL STROKE** | | | | | | | **NON-FATAL STROKE** | | | | | |
| --- | --- | --- | --- | --- | --- | --- | --- | --- | --- | --- | --- | --- | --- |
|  | **MEN** | | | | **WOMEN** | | | **MEN** | | | **WOMEN** | | |
|  | **RR** | | **CI95%** | | **RR** | **CI95%** | | **RR** | **CI95%** | | **RR** | **CI95%** | |
| **Precarious employment trajectories** |  | |  |  |  |  |  |  |  |  |  |  |  |
| ***Constant non-PE*** | ref | | | | ref | | | ref | | | ref | | |
| ***From non-PE to PE*** | 1·00 | | 0·81 | 1·22 | 0·90 | 0·66 | 1·21 | 1·03 | 0·97 | 1·09 | 1·04 | 0·96 | 1·13 |
| ***Constant borderline PE*** | 1·32 | | 1·16 | 1·50 | 1·12 | 0·97 | 1·30 | 1·12 | 1·08 | 1·17 | 1·06 | 1·02 | 1·11 |
| ***Constant precarious*** | 1·21 | | 0·95 | 1·55 | 1·03 | 0·77 | 1·39 | 1·24 | 1·16 | 1·33 | 1·04 | 0·95 | 1·13 |
| **Contractual relation insecurity trajectories** |  | |  |  |  |  |  |  |  |  |  |  |  |
| ***Constant directly employed*** | ref | | | | ref | | | ref | | | ref | | |
| ***Former agency employed*** | 1·02 | 0·38 | | 2·72 | 0·72 | 0·18 | 2·90 | 1·35 | 1·05 | 1·74 | 1·05 | 0·75 | 1·47 |
| **Temporariness trajectories** |  |  | |  |  |  |  |  |  |  |  |  |  |
| ***Constant not temporary*** | ref | | | | ref | | |  | | |  | | |
| ***From temporary to not temporary*** | 0·96 | | 0·84 | 1·11 | 0·91 | 0·74 | 1·12 | 1·03 | 0·99 | 1·07 | 0·99 | 0·93 | 1·05 |
| ***From not temporary to temporary*** | 1·03 | | 0·89 | 1·19 | 0·93 | 0·75 | 1·16 | 0·95 | 0·91 | 0·99 | 0·96 | 0·90 | 1·03 |
| **Multiple job holding trajectories** |  | |  |  |  |  |  |  |  |  |  |  |  |
| ***Constant not MJH*** | ref | | | | ref | | | ref | | | ref | | |
| ***Constant MJH*** | 0·91 | | 0·61 | 1·36 | 0·99 | 0·55 | 1·76 | 1·06 | 0·95 | 1·18 | 1·06 | 0·90 | 1·25 |
| **Income level trajectories** |  | |  |  |  |  |  |  |  |  |  |  |  |
| ***Constant good income*** | ref | | | | ref | | |  | | |  | | |
| ***Constant high income*** | 0·84 | | 0·74 | 0·95 | 1·09 | 0·87 | 1·37 | 0·90 | 0·87 | 0·94 | 0·99 | 0·93 | 1·05 |
| ***Constant low income*** | 1·43 | | 1·22 | 1·68 | 1·25 | 1·07 | 1·45 | 1·19 | 1·13 | 1·26 | 1·09 | 1·04 | 1·14 |
| ***Constant very low income*** | 1·41 | | 0·98 | 2·03 | 1·27 | 1·00 | 1·61 | 1·17 | 1·03 | 1·32 | 1·08 | 1·00 | 1·17 |
| **Collective bargaining agreement trajectories** |  | |  |  |  |  |  |  |  |  |  |  |  |
| ***Constant high CBA*** | ref | | | | ref | | | ref | | | ref | | |
| ***From low to high CBA*** | 1·12 | | 0·78 | 1·59 | 1·22 | 0·63 | 2·38 | 1·03 | 0·93 | 1·15 | 0·98 | 0·78 | 1·21 |
| ***From high to low CBA*** | 0·72 | | 0·50 | 1·05 | 0·85 | 0·55 | 1·31 | 1·13 | 1·04 | 1·24 | 0·99 | 0·88 | 1·12 |
| ***Constant low CBA*** | 0·91 | | 0·70 | 1·19 | 0·57 | 0·31 | 1·07 | 1·00 | 0·93 | 1·08 | 1·04 | 0·90 | 1·20 |

Note: RR are adjusted for continuous age, educational level, family composition, country of birth, purchased medication for treating diabetes, hypertension and dyslipidaemia during exposure measurement, parental socio-economic status of the parents during childhood of study participants and contractual relation insecurity, temporariness, multiple job holding, income level and probability of coverage by collective bargaining agreements trajectories.

Abbreviations: PE (Precarious Employment), MJH (multiple job holders), CBA (collective bargaining agreement).

**Table S8. Adjusted risk ratios for myocardial infarction according to precarious employment component trajectories and income levels at baseline.**

|  | **MEN** | | | | | | **WOMEN** | | | | | |
| --- | --- | --- | --- | --- | --- | --- | --- | --- | --- | --- | --- | --- |
|  | **High income levels** | | | **Low income levels** | | | **High income levels** | | | **Low income levels** | | |
|  | **RR** | **CI95%** | | **RR** | **CI95%** | | **RR** | **CI95%** | | **RR** | **CI95%** | |
| **Contractual relation insecurity trajectories** |  |  |  |  |  |  |  |  |  |  |  |  |
| ***Constant directly employed*** | ref | | | ref | | | ref | | | ref | | |
| ***Former agency employed*** | 1·18 | 0·79 | 1·77 | 0·80 | 0·60 | 1·08 | 1·10 | 0·46 | 2·66 | 1·19 | 0·83 | 1·72 |
| **Temporariness trajectories** |  |  |  |  |  |  |  |  |  |  |  |  |
| ***Constant not temporary*** | ref | | | ref | | | ref | | | ref | | |
| ***From temporary to not temporary*** | 0·95 | 0·90 | 1·00 | 1·03 | 0·99 | 1·07 | 0·98 | 0·83 | 1·17 | 0·91 | 0·85 | 0·98 |
| ***From not temporary to temporary*** | 0·99 | 0·93 | 1·05 | 0·98 | 0·94 | 1·02 | 1·08 | 0·90 | 1·29 | 0·89 | 0·83 | 0·96 |
| **Multiple job holding trajectories** |  |  |  |  |  |  |  |  |  |  |  |  |
| ***Constant not MJH*** | ref | | | ref | | | ref | | | ref | | |
| ***Constant MJH*** | 1·05 | 0·92 | 1·20 | 1·02 | 0·91 | 1·14 | 0·78 | 0·51 | 1·18 | 1·14 | 0·93 | 1·38 |
| **Collective bargaining agreement trajectories** |  |  |  |  |  |  |  |  |  |  |  |  |
| ***Constant high CBA*** | ref | | | ref | | | ref | | | ref | | |
| ***From low to high CBA*** | 1·07 | 0·92 | 1·24 | 1·04 | 0·94 | 1·14 | 0·61 | 0·27 | 1·37 | 0·80 | 0·61 | 1·05 |
| ***From high to low CBA*** | 1·07 | 0·92 | 1·24 | 1·00 | 0·92 | 1·09 | 0·91 | 0·60 | 1·39 | 1·01 | 0·88 | 1·15 |
| ***Constant low CBA*** | 1·03 | 0·90 | 1·18 | 1·02 | 0·96 | 1·09 | 0·63 | 0·33 | 1·22 | 1·01 | 0·86 | 1·18 |

Note: RR are adjusted for continuous age, educational level, family composition, country of birth, purchased medication for treating diabetes, hypertension and dyslipidaemia during exposure measurement, parental socio-economic status of the parents during childhood of study participants and contractual relation insecurity, temporariness, multiple job holding and probability of coverage by collective bargaining agreements trajectories.

Abbreviations: MJH (multiple job holders), CBA (collective bargaining agreement).

**Table S9. Adjusted risk ratios for stroke according to precarious employment component trajectories and income levels at baseline.**

|  | **MEN** | | | | | | **WOMEN** | | | | | |
| --- | --- | --- | --- | --- | --- | --- | --- | --- | --- | --- | --- | --- |
|  | **High income levels** | | | **Low income levels** | | | **High income levels** | | | **Low income levels** | | |
|  | **RR** | **CI95%** | | **RR** | **CI95%** | | **RR** | **CI95%** | | **RR** | **CI95%** | |
| **Contractual relation insecurity trajectories** |  |  |  |  |  |  |  |  |  |  |  |  |
| ***Constant directly employed*** | ref | | | ref | | | ref | | | ref | | |
| ***Former agency employed*** | 1·02 | 0·60 | 1·72 | 1·46 | 1·10 | 1·92 | 0·92 | 0·41 | 2·06 | 1·03 | 0·72 | 1·47 |
| **Temporariness trajectories** |  |  |  |  |  |  |  |  |  |  |  |  |
| ***Constant not temporary*** | ref | | | ref | | | ref | | | ref | | |
| ***From temporary to not temporary*** | 1·02 | 0·95 | 1·08 | 1·03 | 0·98 | 1·08 | 1·01 | 0·88 | 1·16 | 0·97 | 0·91 | 1·03 |
| ***From not temporary to temporary*** | 1·01 | 0·94 | 1·09 | 0·93 | 0·88 | 0·98 | 0·93 | 0·79 | 1·09 | 0·96 | 0·90 | 1·04 |
| **Multiple job holding trajectories** |  |  |  |  |  |  |  |  |  |  |  |  |
| ***Constant not MJH*** | ref | | | ref | | | ref | | | ref | | |
| ***Constant MJH*** | 1·03 | 0·88 | 1·22 | 1·07 | 0·94 | 1·23 | 1·06 | 0·79 | 1·42 | 1·07 | 0·89 | 1·28 |
| **Collective bargaining agreement trajectories** |  |  |  |  |  |  |  |  |  |  |  |  |
| ***Constant high CBA*** | ref | | | ref | | | ref | | | ref | | |
| ***From low to high CBA*** | 1·08 | 0·90 | 1·29 | 1·04 | 0·93 | 1·18 | 1·15 | 0·70 | 1·88 | 0·97 | 0·78 | 1·22 |
| ***From high to low CBA*** | 1·04 | 0·86 | 1·25 | 1·14 | 1·03 | 1·26 | 0·87 | 0·59 | 1·25 | 1·01 | 0·89 | 1·12 |
| ***Constant low CBA*** | 1·03 | 0·87 | 1·22 | 1·05 | 0·96 | 1·13 | 1·42 | 0·98 | 2·06 | 0·96 | 0·83 | 1·12 |

Note: RR are adjusted for continuous age, educational level, family composition, country of birth, purchased medication for treating diabetes, hypertension and dyslipidaemia during exposure measurement, parental socio-economic status of the parents during childhood of study participants and contractual relation insecurity, temporariness, multiple job holding and probability of coverage by collective bargaining agreements trajectories.

Abbreviations: MJH (multiple job holders), CBA (collective bargaining agreement).

**Table S10. Adjusted risk ratios for myocardial infarction according to precarious employment trajectories and income levels at baseline.**

|  | **MEN** | | | | | | | | | **WOMEN** | | | | | | | | |
| --- | --- | --- | --- | --- | --- | --- | --- | --- | --- | --- | --- | --- | --- | --- | --- | --- | --- | --- |
|  | **high income** | | | **medium income** | | | **low income** | | | **high income** | | | **medium income** | | | **low income** | | |
|  | **RR** | **95%CI** | | **RR** | **95%CI** | | **RR** | **95%CI** | | **RR** | **95%CI** | | **RR** | **95%CI** | | **RR** | **95%CI** | |
| **Precarious employment trajectories** |  |  |  |  |  |  |  |  |  |  |  |  |  |  |  |  |  |  |
| ***Constant non-PE*** | ref | | | ref | | | ref | | | ref | | | ref | | | ref | | |
| ***From non-PE to PE*** | 1·04 | 0·89 | 1·21 | 0·98 | 0·93 | 1·04 | 0·90 | 0·80 | 1·01 | 1·09 | 0·70 | 1·69 | 0·95 | 0·84 | 1·09 | 0·86 | 0·74 | 0·99 |
| ***Constant borderline PE*** | 1·01 | 0·93 | 1·10 | 1·03 | 0·99 | 1·07 | 1·12 | 1·02 | 1·24 | 1·09 | 0·86 | 1·39 | 0·96 | 0·89 | 1·04 | 0·92 | 0·83 | 1·01 |
| ***Constant precarious*** | 0·87 | 0·65 | 1·16 | 1·05 | 0·96 | 1·15 | 1·14 | 1·02 | 1·27 | 0·74 | 0·31 | 1·78 | 1·18 | 0·98 | 1·43 | 0·86 | 0·76 | 0·99 |

**Table S11. Adjusted risk ratios for stroke according to precarious employment trajectories and income levels at baseline.**

|  | **MEN** | | | | | | | | | **WOMEN** | | | | | | | | |
| --- | --- | --- | --- | --- | --- | --- | --- | --- | --- | --- | --- | --- | --- | --- | --- | --- | --- | --- |
|  | **high income** | | | **medium income** | | | **low income** | | | **high income** | | | **medium income** | | | **low income** | | |
|  | **RR** | **95%CI** | | **RR** | **95%CI** | | **RR** | **95%CI** | | **RR** | **95%CI** | | **RR** | **95%CI** | | **RR** | **95%CI** | |
| **Precarious employment trajectories** |  |  |  |  |  |  |  |  |  |  |  |  |  |  |  |  |  |  |
| ***Constant non-PE*** | ref | | | ref | | | ref | | | ref | | | ref | | | ref | | |
| ***From non-PE to PE*** | 1·01 | 0·83 | 1·22 | 0·99 | 0·93 | 1·07 | 0·93 | 0·82 | 1·06 | 1·08 | 0·75 | 1·56 | 0·99 | 0·88 | 1·12 | 1·00 | 0·87 | 1·14 |
| ***Constant borderline PE*** | 1·02 | 0·92 | 1·14 | 1·04 | 0·99 | 1·09 | 1·13 | 1·01 | 1·25 | 0·99 | 0·81 | 1·22 | 0·99 | 0·93 | 1·07 | 1·00 | 0·91 | 1·10 |
| ***Constant precarious*** | 0·99 | 0·71 | 1·39 | 1·28 | 1·16 | 1·43 | 1·04 | 0·92 | 1·18 | 0·83 | 0·41 | 1·65 | 0·95 | 0·78 | 1·16 | 0·98 | 0·86 | 1·11 |
